# Selected Markers of Inflammation in the Saliva of Children Infected with *Helicobacter pylori*

**DOI:** 10.3390/ijms252312780

**Published:** 2024-11-28

**Authors:** Mateusz Zakrzewski, Agnieszka Gornowicz, Magdalena Zakrzewska, Anna Bielawska, Elżbieta Maciorkowska

**Affiliations:** 1 Department of Urology and Oncological Urology, Voivodeship Hospital in Lomza, 18-404 Lomza, Poland; mateusz.zakrzewski4@wp.pl; 2 Department of Biotechnology, Medical University of Bialystok, 15-089 Bialystok, Poland; anna.bielawska@umb.edu.pl; 3 Department of Pediatrics, Gastroenterology, Hepatology, Nutrition, Allergology and Pulmonology, Medical University of Bialystok, 15-274 Bialystok, Poland; magdalena.maciorkowska@gmail.com; 4 Department of Developmental Age Medicine and Pediatric Nursing, Medical University of Bialystok, 15-295 Bialystok, Poland; emaciorkowska@o2.pl

**Keywords:** salivary markers, inflammation, *Helicobacter pylori*, cytokines, metalloproteinases, TLR-2, calprotectin, defensins

## Abstract

*Helicobacter pylori* has been of interest to scientists and clinicians for many years, often causing diagnostic difficulties, especially in the youngest age group, in children. The presence of this bacterium in the population depends on the geographic region. However, it is assumed that even half of the world’s population may be infected with *H. pylori*. Children infected with *H. pylori*—the study group (Hp(+)) and control group (Hp(−)), were chosen for further examination. The aim of the study was to analyze the concentrations of selected inflammatory markers in saliva (TNF-α, IL-8) and other markers (neutrophil defensin-1, sICAM-1, calprotectin, metalloproteinase-9, metalloproteinase-2, lactotransferrin, TLR-2) using ELISA technique. We confirmed the increased concentrations of IL-8, ND-1, and TLR-2 in the group of children infected with *Helicobacter pylori*. Moreover, there was also a positive, significant correlation between the concentration of ND-1 and MMP-2, sICAM-1, and calprotectin as well as MMP-9 and MMP-2 in the group of infected children. The study created new possibilities of insight into the pathogenetic mechanisms of developing inflammation in the mouth. This type of comprehensive research is also used to monitor the current disease process and create new opportunities for better in-depth diagnostics of children infected with *H. pylori*.

## 1. Introduction

*Helicobacter pylori* was recognized in 1994 as a type I carcinogen for gastric cancer and the second-leading cause of cancer-related deaths worldwide [1]. Moreover, the bacterium causes a number of ailments coming from the gastrointestinal tract, called dyspeptic symptoms. However, not all patients develop gastric symptoms. In some cases, the existence of bacteria is silent, or manifests itself when immunity decreases, or it becomes a trigger for other diseases located outside the stomach, e.g., asthma, ischemic heart disease, non-alcoholic fatty liver disease, type 2 diabetes mellitus, celiac disease, caries, inflammatory bowel disease, as well as multiple sclerosis, and some skin diseases, e.g., vitiligo, chronic urticaria, psoriasis, alopecia areata, rosacea, primary immune thrombocytopenia, anemia, Henoch–Schonlein purpura, and many others [2,3,4,5,6,7,8,9,10,11,12]. Invasive diagnosis of *Helicobacter pylori* involves gastroscopy, during which biopsies are taken from the gastric mucosa, then assessed in a histological examination and a rapid urease test [13]. Non-invasive diagnostics are constantly being developed, and the most important tests include the urea breath test and the detection of *Helicobacter pylori* antibodies in blood and stool [14,15]. Saliva is becoming a valuable material for non-invasive diagnostics. It represents a valuable diagnostic tool in many diseases, such as diabetes, hepatitis, oral diseases, and immunodeficiency disease. Water is the major component of saliva and is responsible for oral clearance. The organic substance is composed of enzymes (lysozyme, amylases, peroxidase, lipase), hormones (cortisol), mucins, and immunoglobulins. Electrolytes act as a buffer and protect teeth [16].

The oral cavity is the first section of the digestive tract where this bacterium may colonize. Molecular tests showed the presence of *Helicobacter pylori* in the oral cavity of people with dyspeptic symptoms and the confirmed presence of *Helicobacter pylori* in biopsies taken during gastroscopy [17,18].

Among patients diagnosed with *Helicobacter pylori* in the oral cavity, severe inflammation of the oral cavity was observed, leading to periodontal disease and caries. There is a known relationship between oral diseases such as periodontitis, recurrent aphthous stomatitis, glossitis, burning mouth syndrome, and the presence of *Helicobacter pylori* in the supra- and subgingival plaque [19].

The aim of the study was to evaluate cytokines (TNF-α, IL-8) and other markers of inflammation (neutrophil defensin-1, sICAM-1, calprotectin, metalloproteinase-9, metalloproteinase-2, lactotransferrin, TLR-2) in the saliva of children with existing, confirmed *Helicobacter pylori* infection gastric mucosa. Assessment of existing correlations in the concentrations of cytokines (TNF-alpha, IL-8) and other inflammatory markers (ND-1, sICAM-1, calprotectin, metalloproteinase-9, metalloproteinase-2, lactotransferrin, TLR-2) in infected children and in the control group will allow us to answer the question whether the presence of *H. pylori* in the gastric mucosa may affect the condition of the teeth and inflammation of the oral mucosa in infected children.

## 2. Results

### 2.1. Characteristic of the Groups

The study included 117 children with dyspeptic symptoms hospitalized in the Department of Paediatrics, Gastroenterology, Hepatology, Nutrition and Allergology, University Children’s Clinical Hospital in Białystok. After conducting research on a group of 117 children and obtaining the results of histopathological examination of samples taken during gastroscopic examination, as well as the results of the urease test, the children were divided into two groups.

The first of them were children with a positive histopathological result confirming *Helicobacter pylori* infection in the gastric mucosa Hp(+). The percentage of Hp(+) children in the study group was 38.5% (N = 45). Girls constituted 55.6% and boys 44.4% of the infected group (Table 1).

**Figure 1:**
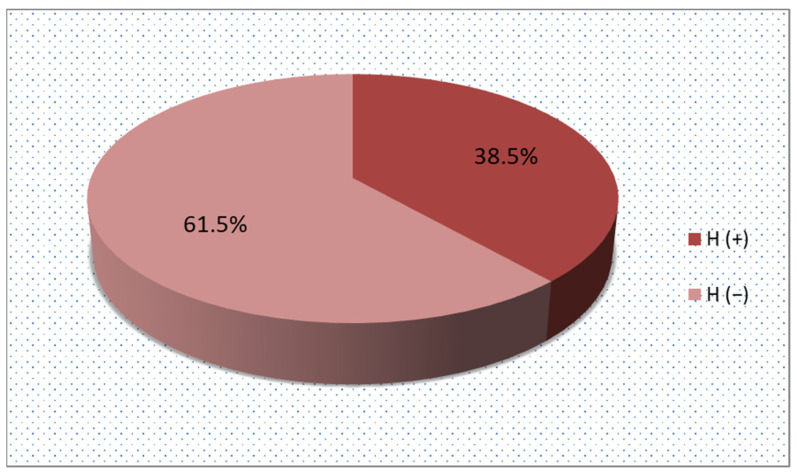
The result of histopathological examination in the study group.

The control group consisted of children with dyspeptic symptoms in whom the presence of *Helicobacter pylori* in the gastric mucosa was not confirmed by gastroscopy. The percentage of children with Hp(−) was 61.5% (N = 72) (Figure 1). Girls constituted 72.2% and boys 27.8%.

The average age of children from the study group with a positive histopathological result of Hp(+) was 13.6 years ± 3.6 years, while in the study group with a negative histopathological result of Hp(−), the mean age was 13.2 years ± 3.2 years (Table 2).

The analysis of the parents’ age in the group of children infected with *Helicobacter pylori* Hp(+) showed that the average age of the mothers was 40.2 years (standard deviation ± 6.1 years). The mean age of the fathers was 44.4 years with a standard deviation of ±7.3 years.

In the group of children with a negative histopathological result of Hp(−), the average age of mothers was 41.8 years (standard deviation ± 5.4 years). The mean age of the fathers was 43.0 years (standard deviation ± 5.0 years) (Table 3).

### 2.2. Characteristics of the Studied Group of Children in Terms of Clinical Symptoms

One of the most common clinical symptoms reported by children in the study group was abdominal pain. The frequency of its occurrence was higher in the group of children without *H. pylori* infection admitted to the Clinic and qualified for gastroscopic examination due to dyspeptic symptoms (90.3%). The percentage of children with abdominal pain in the Hp(+) infected group was lower and amounted to 84.4%.

Loss of appetite as a symptom coexisting with abdominal pain in children in the Hp(+) group was observed in 25 subjects, which constituted 55.6% of the study group, compared to the Hp(−) group, in which this percentage was slightly lower and amounted to 48.6%, which was a positive response in 35 children from this group.

The causal effect of pain in the abdominal cavity and the resulting loss of appetite was a decrease in body weight, which was manifested in practically the same percentage in both groups. In the Hp(+) group, a weight loss was observed in 13 subjects, which was 28.9%, compared to the Hp(−) group, where a weight loss was observed in 21 subjects, which was 29.2% in this group.

The study also compared symptoms outside the gastrointestinal tract that may coexist with *Helicobacter pylori* infection. As we know, during infection with this bacterium, some people, including children, may experience skin reactions of varying intensity, e.g., vitiligo, urticaria, psoriasis, alopecia, rosacea, as well as other diseases outside the gastrointestinal tract, such as primary immune thrombocytopenia, anaemia, Henoch–Schönlein purpura, and others.

Among the examined patients, significant data concerned only the symptoms of anaemia and the occurrence of urticaria. Anaemia was observed much more often in the group of infected children (28.9%) than in the group of children with dyspeptic symptoms without *H. pylori* infection (15.3%). An almost identical percentage of urticaria was reported in the examined children. Data on the frequency of clinical symptoms in the study group are presented in Table 4.

### 2.3. Assessment of the Concentration of Selected Inflammatory Markers in the Saliva of the Examined Children

The assessment of the concentrations of selected inflammatory markers in the saliva in a group of children infected with *Helicobacter pylori* Hp(+) compared to the control group Hp(−) was the aim of the conducted research.

It is assumed that cytokines play an important role in the pathogenesis of gastrointestinal inflammation caused by *H. pylori* infection, in particular TNFα and IL-8. Moreover, the assessment of the concentration of these cytokines in unstimulated saliva was undertaken mainly due to the fact that there was a significant increase in the concentration of these cytokines in gastric mucosa homogenates and in gastric juice in children infected with this bacterium. IL-8 is assigned a significant role in the development of gastritis. The assessment of IL-8 concentration in the saliva in children infected with *H. pylori* Hp(+) was 378.8 pg/mL (±149.8), while in the Hp(−) control group the concentration of the cytokine was 282.4 pg/mL (±163.2). These values differed statistically significantly (*p* = 0.032).

The mean concentration of TNFα in the saliva assessed in the group of infected children was 14.7 pg/mL (±8.8) and in the Hp(−) group it was 16.1 pg/mL (±8.9).

We also detected sICAMCD54 concentration in both examined groups. The mean value was not statistically different between the Hp(+) and Hp(−) groups.

The average sICAMCD54 concentration in the saliva of infected children was 11.5 pg/mL (±6.3), and in the uninfected group it was 12.4 pg/mL (±7.9). Detailed data is presented in Table 5.

Numerous processes occurring at the cellular level show that the activity of metalloproteinase enzymes is an inherent factor in the inflammatory reaction. *Helicobacter pylori* infection induces the migration of pro-inflammatory factors, a local and systemic response to the pathogen, causing inflammation and an increased concentration of pro-inflammatory molecules and cells. The study assessed the concentration of metalloproteinase-2 and metalloproteinase-9 in the inflammatory response to *Helicobacter pylori* due to the most detailed knowledge of these molecules, as well as their specificity in infection with this pathogen.

The average concentration of both MMP-9 and MMP-2 assessed in unstimulated saliva in both groups of studied children was comparable. Detailed data is presented in Table 6.

Neutrophil defensins and lactotransferrin are innate immune response proteins produced by neutrophils and the gastrointestinal tract as a result of pathogen invasion. Defensins are granules secreted mainly by neutrophils, as well as by most epithelial cells, including the epithelial cells of the gastrointestinal tract, which have a strong phagocytic effect against bacteria, fungi, and viruses.

The concentration of neutrophil defensin-1 (ND-1) assessed in unstimulated saliva of children infected with Hp(+) was 50.6 pg/mL (±47.1), while in the group of children with Hp(−) it differed statistically significantly compared to the group of infected children and the concentration was 38.3 pg/mL (±62.7) (*p* < 0.020).

The concentration of lactotransferrin assessed in unstimulated saliva of children infected with Hp(+) was 0.2 pg/mL (±0.6), while in the group of children with Hp(−) the concentration reached 0.5 pg/mL (±2.0).

The concentration of calprotectin in the saliva in both groups of children was low and in the group of infected children it was 91.4 pg/mL (±61.4), and in the group of children without *H. pylori* infection it was 119.2 pg/mL (±156.0) (Table 7).

Laboratory studies have shown that Toll-like receptors, in particular TLR-2, TLR-4, and TLR-9, are most important for the recognition of molecular patterns associated with *Helicobacter pylori* infection. TLR-2 is the most numerous group of receptors tested in response to *Helicobacter pylori* infection in epithelial cells of the gastrointestinal tract.

The average TLR2 concentration assessed in unstimulated saliva of children infected with *Helicobacter pylori* was 4.1 pg/mL (±6.0), while in the group without infection it was significantly lower and amounted to 0.7 pg/mL (±0.4) (*p* < 0.001). Detailed data are presented in Table 8.

**Figure 2:**
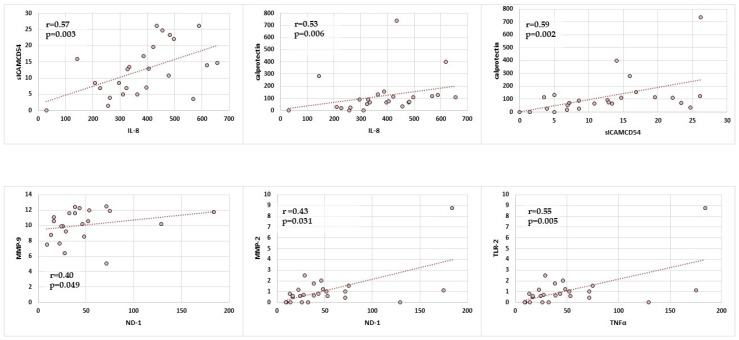
Correlations between concentrations of selected inflammatory markers in the saliva of children Hp(−). Points on the scatterplot—data of study participants; line on the scatterplot—univariate linear regression.

Next, correlations between selected markers associated with inflammation in the saliva of children without *Helicobacter pylori* infection were analysed, and statistically significant positive correlations are presented in Figure 2.

In the group of children without *H. pylori* infection, a statistically significant positive correlation between IL-8 and sICAM-1 was found in unstimulated saliva (r = 0.57; *p* = 0.003). Statistically significant positive correlations were also shown for the concentrations of calprotectin and IL-8 in saliva samples. Furthermore, statistically significant positive correlations were demonstrated between the salivary concentrations of calprotectin and sICAM-1. However, statistically significant positive correlations were found between the concentrations of MMP-2 or MMP-9 and ND-1. Finally, statistically significant positive correlations were demonstrated between TLR-2 and TNF-α.

**Figure 3:**
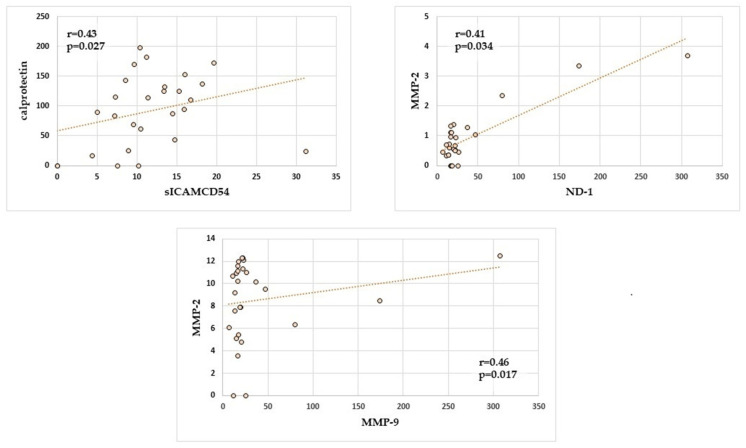
Correlations between concentrations of selected inflammatory markers in the saliva of children Hp(+). Points on the scatterplot—data of study participants; line on the scatterplot—uni-variate linear regression.

Next, correlations between selected markers associated with inflammation in the saliva of children with *Helicobacter pylori* infection were analysed, and statistically significant positive correlations are presented in Figure 3.

In the group of children infected with *Helicobacter pylori*, there was a statistically significant (*p* = 0.017) and positive correlation (r = 0.46) between MMP-2 and MMP-9 in the unstimulated saliva in the examined children. Additionally, statistically significant positive correlations were found between calprotectin and sICAM-1 as well as MMP-2 and ND-1.

## 3. Discussion

Chronic infection with *Helicobacter pylori* induces an immune response, which leads to the mediation of inflammatory cells and their secretion of pro-inflammatory compounds, which contribute to the chronic inflammatory process of the gastric mucosa, duodenum, and oral cavity. The bacteria living in the oral cavity, in sub- and supragingival pockets, creates an unfavorable biofilm on the surface of teeth. This process causes the spread of bacteria throughout the oral cavity and also leads to reinfection of the gastrointestinal tract, causing periodically recurring dyspeptic symptoms along with the occurrence of gastric and duodenal ulcers, and is also an important factor in carcinogenesis in adults with long-term exposure [20,21,22].

The aim of our study was to assess the concentrations of selected inflammatory markers in the saliva of children infected with *Helicobacter pylori*, as well as to compare the concentrations of the tested markers in the saliva of the examined children depending on the clinical symptoms.

The inflammatory process caused by *Helicobacter pylori* activates specific inflammatory mediators that participate in the immune response, stimulating the entire system. Previous research clearly indicates specific groups and pro-inflammatory substances. In the group of cytokines, IL-1β, IL-6, IL-8, IL-18, TNFα, IFNγ, as well as the adhesion molecules ICAM-1, play an important role [23,24,25,26].

In our own work, the concentrations of selected cytokines that seemed the most promising were tested. The obtained saliva samples were tested for TNFα, IL-8, and sICAM-1 (CD54) concentrations.

The IL-8 concentration in the saliva in children infected with *Helicobacter pylori* was higher compared to the control group, as shown by mean concentration of 378.8 pg/mL (±149.8) and 282.4 pg/mL (±163.2), respectively. The values differed statistically significantly (*p* = 0.032). TNF-α and sICAM-1 results were similar in the study group and the control group. No statistical significance was demonstrated in both study groups.

Further statistical analysis showed positive correlations between inflammatory markers in saliva. The correlation between sICAM-1 and IL-8 concentrations showed a statistically significant difference between IL-8 and sICAM-1 concentrations in the group of children with Hp(−).

As research by Sahibzada et al. showed, IL-8, IL-6, and TNFα cytokines assessed in saliva are good markers of the oral inflammatory response in saliva. In experimental studies, it is also used to monitor neogenesis in people after oral cancer treatment [25]. Elevated values of pro-inflammatory cytokines IL-6, IL-8, and TNFα in saliva are found in people with dental caries and chronic stomatitis [23].

The studies carried out so far on enzymes specific for the response against *Helicobacter pylori* indicate that the most important function is played by metalloproteinases-2, -8, -9. Enzymes that are in an inactive form inside the cell are activated extracellularly, and their activation is driven by many mechanisms, including the process of cellular transcription and translation, as well as the activity of specific tissue inhibitors [27,28].

In our study, it was decided to measure the concentration of metalloproteinase-2 and -9. Their role is closely related to each other, and the concentration of metalloproteinase-2 induces an increase in metalloproteinase-9. Moreover, metalloproteinases are involved in signal transduction for cytokines and chemokines, and also play an important role in angiogenesis.

The concentration of both metalloproteinase-9 and metalloproteinase-2 assessed in unstimulated saliva in both groups of studied children was comparable. No statistical significance was demonstrated for the tested concentrations of metalloproteinases in both groups.

However, there was a positive, statistically significant correlation between the concentrations of metalloproteinase-9 and metalloproteinase-2 in the saliva in children infected with *Helicobacter pylori*.

Nevertheless, James et al. proved in in vitro studies that there is a close relationship between *H. pylori* infection and the production and secretion of metalloproteinase-9 by macrophages, which contributes to the local inflammatory response [29].

The cellular response against *Helicobacter pylori* is based on substances secreted by granulocytes, which play an important role in human immunity. Specific substances such as defensins (α- and β- defensins), lactotransferrin, and calprotectin are secreted to fight against bacterial infection [30,31,32]. In the next step of our study, the concentrations of neutrophil defensin-1, lactotransferrin, and calprotectin in saliva were examined.

The ND-1 concentration assessed in unstimulated saliva of children infected with *Helicobacter pylori* was significantly higher compared to the control group it. However, there was no statistical significance between lactotransferrin concentration assessed in the saliva in infected children in comparison with children without infection, but it was slightly higher.

Both neutrophil defensins and lactotransferrin are the main compounds secreted by neutrophils during the immune response. Their role is primarily to enhance phagocytosis.

Neutrophil defensins are proteins secreted by neutrophil granules during the immune response. The presence of numerous neutrophils in the gastrointestinal tract plays an important role in the fight against microorganisms entering the food [33]. It has been proven that both α-defensins and β-defensins are produced in large numbers by neutrophils during the response against *Helicobacter pylori.* Researchers observed significantly higher concentrations of neutrophil defensins in gastric juice during inflammatory diseases of the gastrointestinal tract, as well as during *Helicobacter pylori* infection [34].

Lactotransferrin is secreted mainly through secretions—milk, tears, saliva, secretions from the nasal epithelium, respiratory tract, digestive tract, reproductive tract, and also from feces, urine, bile, and others. Lactotransferrin has a high affinity for iron ions. The structure of this protein contains an iron ion, which leads to conformational changes in the lactotransferrin molecule and enables its killing effect. Anaemia occurring significantly in the examined children infected with *Helicobacter pylori* compared to the control group may have two causes [35,36]. Iron ions are captured by inflammatory mediators, including lactotransferrin, for increased synthesis,; hence, the depletion of stores in the chronic inflammatory process of the gastric mucosa, esophagus, oral cavity, and other parts of the digestive tract caused by *Helicobacter pylori*. On the other hand, the lack of iron ions in the examined patients infected with *Helicobacter pylori* and accompanied by anaemia could have resulted in insufficient synthesis of lactotransferrin and its low concentrations noted in our study [37].

The concentration of calprotectin in the saliva in both groups of children was low. We detected 91.4 pg/mL (±61.4) and 119.2 pg/mL (±156.0) in the group of children without *H. pylori* infection. There was no statistical significance for calprotectin concentrations between the groups.

Moreover, in the studied group of children infected with *Helicobacter pylori*, there was also a positive, statistically significant correlation between the concentration of sICAM-1 and calprotectin in saliva.

Calprotectin plays an important role in the inflammatory process. It intensifies inflammatory processes, enhances intercellular signals, is responsible for cell apoptosis, and supports the migration, adhesion, and phagocytosis of neutrophils. As evidenced by Dhas, signal transduction for calprotectin occurs through Toll-like receptors [32]. The authors emphasize the role of the TLR-4 receptor. However, the available literature also confirms the relationship of calprotectin with the activation of TLR-2 and TLR-9 receptors [38,39].

Toll-like receptors are involved to the immune response against *H. pylori*. The most important of them and the best studied so far are TLR-2, TLR-4, and TLR-9 receptors [40,41,42,43]. Moreover, TLR-2 and TLR-4 receptors show significant activity in epithelial cells during stomatitis, as well as periodontitis [44].

In our own study, the concentration of the TLR-2 receptor in the saliva in children infected with *Helicobacter pylori* was assessed. The average TLR-2 concentration assessed in unstimulated saliva of children infected with *Helicobacter pylori* was 4.1 pg/mL (±6.0), while in the group without infection it was much lower and amounted to 0.7 pg/mL (±0.4). Statistical significance was demonstrated (*p* < 0.001).

*H. pylori* was suggested to be responsible for a number of extra-gastric manifestations. In particular, it was shown that anaemia was observed much more often in the group of children with Hp(+) than in the group of children without *H. pylori* infection. Our study also revealed that IL-8, ND-1, as well as TLR-2 play the most significant role in children infected with *Helicobacter pylori*. Moreover, there was also a positive, significant correlation between the concentration of neutrophilic defensin-1 and metalloproteinase-2 in the group of infected children. A positive, significant correlation was also found between sICAM-1 and calprotectin in the group of infected children. Additionally, we observed a statistically significant positive correlation between metalloproteinase-9 and metalloproteinase-2 in the group of infected children.

## 4. Materials and Methods

The study was approved by the Bioethics Committee of the Medical University of Bialystok, and consent was obtained for its conduct (no. R-I-002/381/2018). Participants received detailed and exhaustive information about the course and purpose of the study, were informed about the possibility of resignation at each stage, and gave their written consent to participate in all components of the study before it began.

The study included 117 children with dyspeptic symptoms hospitalized in the Department of Paediatrics, Gastroenterology, Hepatology, Nutrition and Allergology, University Children’s Clinical Hospital in Bialystok. The mean age for the examined group of children was 13.3 ± 3.4 years. The mean age of boys was 11.8 ± 3.5 years (minimum 4.6 years, maximum 17.6 years). The mean age of the girls was 14.1 years ± 3.1 years (minimum 6.3 years, maximum 17.8 years). The control group consisted of children with dyspeptic symptoms in whom the presence of *Helicobacter pylori* in the gastric mucosa was not confirmed by gastroscopy.

### 4.1. Collection of Saliva Samples from the Examined Children

Saliva was collected using a standard saliva-collection method between 9:00 a.m. and 11:00 a.m. All subjects refrained from eating, drinking, and brushing their teeth for at least 2 h before sample collection. Unstimulated saliva was collected into tubes by spitting method for a minimum of 10 min. Then the samples were homogenized and centrifuged at 1200 rpm for 15 min at 4 °C. The samples were portioned, frozen, and stored at −80 °C until the next stage of research was implemented. The methodology was presented in our previous paper [45].

### 4.2. Determination of the Concentration of TNF-α, IL-8, sICAM-1, Metalloproteinases (MMP-2, MMP-9), Lactotransferrin, Calprotectin, ND-1, TLR-2 in the Saliva of Examined Children

High-sensitivity ELISA kits from EIAab were used to determine the concentration of proteins in the tested saliva samples. The plate was pre-coated with an antibody specific to the tested antigen. The examined samples, blank, and standards were applied to the plate using an automatic pipette. Then, after 120 min of incubation at 37 °C, the plate was incubated with the antibody combined with biotin for 60 min at 37 °C. The next step was to remove the contents of the plate wells and wash them three times with washing buffer, followed by incubation with avidin combined with Horseradish Peroxidase. After completing the above steps, tetramethylbenzidine (TMB) substrate was added to each well of the plate using an automatic pipette. The wells in which the reaction occurred contained the test substance and changed their color depending on the concentration of antigen in the sample. The reaction was terminated by adding sulfuric acid. In the next stage of the study, spectrophotometric measurement was performed at a wavelength of 450 nm ± 2. The results of the wavelength measurement were compared with a standardized calibration curve by reading the antigen concentration results for individual samples tested. The results are presented in pg/mL and showed in the Tables.

### 4.3. Statistical Analysis

Statistical analysis was performed using STATISTICA 12 software (StatSoft, Cracow, Poland). The significance level was *p* < 0.05. The values of the median, as well as the min. and max., were determined.

## 5. Conclusions

Despite significant progress in the field of non-invasive diagnosis of *Helicobacter pylori* infection, the results of research still leave many questions, and scientists are looking for more and more sensitive and specific diagnostic methods for the presence of bacteria, its metabolites, or predictors that could indicate about ongoing inflammation in the gastrointestinal tract resulting from the presence of *Helicobacter pylori* there. It is also necessary to be aware that the ongoing disease process in the oral cavity from the period of developmental age is always individually differentiated and should be related to specific biomarkers. Testing their concentration in saliva during persistent *Helicobacter pylori* infection has created new opportunities for insight into the pathogenetic mechanisms of developing inflammation. Conducting further comprehensive research will contribute to a better understanding of the nature of the disease process and will provide insight into the dynamics of the disease. This type of comprehensive research also serves to monitor the current disease process and create new opportunities for better, in-depth diagnosis of infected children.

## Figures and Tables

**Table 1:** Characteristics of the study group of children in terms of gender.

Tested Children	Group Size		Girls		Boys	
	N	%	N	%	N	%
Children with *H. pylori*	45	38.5	25	55.6	20	44.4
Control group	72	61.5	52	72.2	20	27.8

**Table 2:** Age of the examined children divided into Hp(+) and Hp(−).

Tested Groups	Group Size	Mean Age	Median	Min.	Max.	SD
Hp(+)	38.5%	13.6	14.8	4.9	17.8	3.6
Hp(−)	61.5%	13.2	13.8	4.6	17.8	3.2

**Table 3:** Age of the parents of the examined children divided into Hp(+) and Hp(−).

			Mean Age in Years				
		Group Size	Mean Age	Median	Min.	Max.	SD
Mother	Hp(+)	44	40.2	41.0	29.0	52.0	6.1
	Hp(−)	72	41.8	41.5	28.0	56.0	5.4
Father	Hp(+)	42	44.4	44.0	30.0	60.0	−7.3
	Hp(−)	66	43.0	43.0	28.0	56.0	−5.0

**Table 4:** Characteristics of clinical symptoms in the studied group of children.

	Tested Children in Individual Groups					
Clinical Symptoms		Hp(+)			Hp(−)	
	N		%	N		%
Abdominal pain	38		84.4	65		90.3
Loss of appetite	25		55.6	35		48.6
Weight loss	13		28.9	21		29.2
Anaemia	13		28.9	11		15.3
Urticaria	6		13.3	10		13.9

**Table 5:** Concentration of selected inflammatory cytokines in the saliva in the examined children.

Tested Marker [pg/mL]	Hp	N	Mean Concentration	SD	Min.	Median	Max.	*p* *
TNFα	(+)	27	14.7	8.8	2.1	12.4	38.3	0.469
	(−)	25	16.1	8.9	2.4	15.5	41.3	
IL-8	(+)	25	378.8	149.8	29.7	387.0	656.0	0.032
	(−)	25	282.4	163.2	0.0	241.0	596.0	
sICAMCD54	(+)	27	11.5	6.33	0.0	10.5	31.2	0.949
	(−)	25	12.4	7.9	0.0	12.7	26.2	

*p* *—statistical significance (*p* < 0.05).

**Table 6:** Concentration of selected metalloproteinases in the saliva in the examined children.

Tested Marker [pg/mL]	Hp	N	Mean Concentration	SD	Min.	Median	Max.	*p* *
MMP-9	(+)	27	8.5	3.6	0.0	9.5	12.5	0.595
	(−)	25	8.9	3.9	0.0	10.2	12.5	
MMP-2	(+)	27	0.9	0.9	0.0	0.7	3.7	0.797
	(−)	25	1.1	1.7	0.0	0.7	8.8	

*p* *—statistical significance (*p* < 0.05).

**Table 7:** Concentration of ND-1, lactotransferrin, and calprotectin in the saliva of the examined children.

Tested Marker [pg/mL]	Hp	N	Mean Concentration	SD	Min.	Median	Max.	*p* *
ND-1	(+)	27	50.6	47.1	8.9	38.3	184.0	0.020
	(−)	25	38.3	62.7	6.8	18.9	307.0	
Lactotransferrin	(+)	27	0.2	0.6	0.0	0.0	2.5	0.908
	(−)	25	0.5	2.0	0.0	0.0	10.1	
Calprotectin	(+)	27	91.4	61.4	0.0	94.3	198.1	0.748
	(−)	25	119.2	156.1	0.0	75.2	737.0	

*p* *—statistical significance (*p* < 0.05).

**Table 8:** TLR-2 receptor concentration in the saliva in the examined children.

Tested Marker [pg/mL]	Hp	N	Mean Concentration	SD	Min.	Median	Max.	*p* *
TLR-2	(+)	27	4.1	6.0	0.0	1.5	20.0	0.000
	(−)	25	0.7	0.4	0.0	0.6	2.1	

*p* *—statistical significance (*p* < 0.05).

## Data Availability

Data are contained within the article.

## Abbreviations

ELISA	Enzyme-linked immunosorbent assay
*H. pylori*	*Helicobacter pylori*
IL-1β	Interleukin-1β
IL-8	Interleukin-8
IL-18	Interleukin-18
IFNγ	Interferon γ
Max.	Maximum
Min.	Minimum
MMP-2	Metalloproteinase-2
MMP-9	Metalloproteinase-9
ND-1	Neutrophil defensin-1
sICAM-1 (CD54)	Soluble intercellular adhesion molecule-1 (CD54)
TLR-2	Toll-like receptor-2
TLR-4	Toll-like receptor-4
TLR-9	Toll-like receptor-9
